# Anisotropic Electron–Phonon Interactions in 2D Lead-Halide Perovskites

**DOI:** 10.1021/acs.nanolett.4c01905

**Published:** 2024-07-08

**Authors:** Jaco J. Geuchies, Johan Klarbring, Lucia Di Virgilio, Shuai Fu, Sheng Qu, Guangyu Liu, Hai Wang, Jarvist M. Frost, Aron Walsh, Mischa Bonn, Heejae Kim

**Affiliations:** † Max Planck Institute for Polymer Research, 55128 Mainz, Germany; ‡ Department of Materials, Imperial College London, London SW7 2AZ, United Kingdom; § Department of Physics, Chemistry and Biology (IFM), Linköping University, SE-581 83 Linköping, Sweden; ∥ Department of Physics, Imperial College London, London SW7 2AZ, United Kingdom; ⊥ Department of Physics, Pohang University of Science and Technology, 37673 Pohang, Korea

**Keywords:** Low-dimensional perovskites, electron−phonon coupling, polarons, ultrafast THz spectroscopy

## Abstract

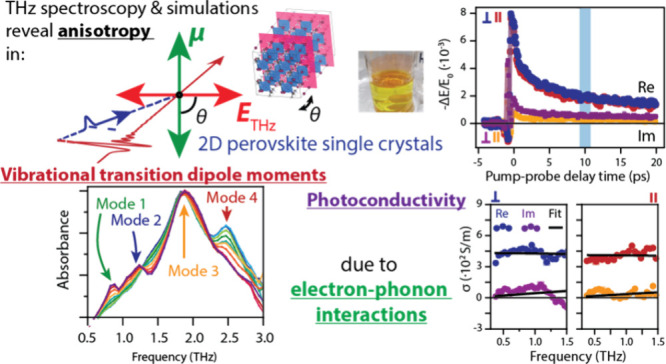

Two-dimensional (2D) hybrid organic–inorganic metal halide perovskites offer enhanced stability for perovskite-based applications. Their crystal structure’s soft and ionic nature gives rise to strong interaction between charge carriers and ionic rearrangements. Here, we investigate the interaction of photogenerated electrons and ionic polarizations in single-crystal 2D perovskite butylammonium lead iodide (BAPI), varying the inorganic lamellae thickness in the 2D single crystals. We determine the directionality of the transition dipole moments (TDMs) of the relevant phonon modes (in the 0.3–3 THz range) by the angle- and polarization-dependent THz transmission measurements. We find a clear anisotropy of the in-plane photoconductivity, with a ∼10% reduction along the axis parallel with the transition dipole moment of the most strongly coupled phonon. Detailed calculations, based on Feynman polaron theory, indicate that the anisotropy originates from directional electron–phonon interactions.

Hybrid lead-halide perovskites are one of the first ionic semiconductors where the diffusion of the photoexcited charge carriers exceeds 1 μm.^1^ Several of the advantageous properties of hybrid perovskites, e.g. reduced carrier-impurity interactions,^2^ long charge carrier lifetimes,^3^ and high defect tolerance^4^ have been associated with the material’s distinctive electron–phonon interactions, which set a limit for the maximum carrier density and mobility in perovskites.^5^ Despite outstanding characteristics, the instability of the halide perovskites in ambient environments has hindered its applicability. Since two-dimensional (2D) perovskites exhibit enhanced stability and enable improved performance of 2*D*/3D heterostructures,^6−9^ it would be ideal to systematically investigate potentially interesting optoelectronic properties arising from the confinement.

The perovskite structure can be transformed from 3D into 2D by replacing some of the smaller A-site cations with longer C-chain organic molecules.^10−13^ These longer spacer molecules push the lattice apart into two-dimensional metal-halide lamellae with tunable thicknesses, altering the energetic landscape of photoexcited carriers due to quantum- and dielectric confinement.^14^ The vibrational properties of low-dimensional perovskites also differ from their 3D counterparts. Due to a large impedance mismatch between the inorganic and organic layers in 2D perovskites, acoustic phonon propagation, is two times slower compared to 3D perovskites.^15^ Butylammonium lead iodide (BAPI) perovskites have been studied extensively,^16−18^ but especially when synthesized through a synthesis protocol involving spin coating,^11,19^ it is hard to directly obtain large-area crystals which are phase pure in terms of the thickness of the inorganic structure *n*. Room-temperature Raman microspectroscopy measurements in literature have revealed in-plane anisotropy of the Raman-active modes,^20^ which were shown to be strongly heterogeneously broadened upon lowering the temperature.^21^

Thermally accessible vibrational modes (up to 25 meV (6 THz) at room temperature) of perovskites originate mostly from displacements of the inorganic framework.^22−25^ Previous studies have shown that these low-frequency phonon modes couple strongly to electronic states around the band extrema of the 3D perovskite.^26^ Specifically, in 3D methylammonium lead iodide (MAPI), the displacement of the structure along the 1 THz phonon coordinate was revealed as the mode that dominates electron–phonon coupling and has been used to explain the temperature dependence of the bandgap of MAPI.^26−28^ Although the exact structure of the polaron in metal halide perovskites is still under debate,^29^ it is clear that coupling between vibrations and charge carriers in these materials impacts their static and dynamic optoelectronic properties.^30,31^

Anisotropic electron mobilities have been observed in, and predicted for, various semiconductor and metallic materials, e.g. TiO_2_,^32,33^ phosphorus carbide,^34^ 2D niobium selenide^35^ and Borophene.^36^ In tetracene crystals, the anisotropy of the electron mobility is directly correlated with the vibrational properties of the material.^37^ While previous studies on 2D perovskites have focused on transport anisotropy between the inorganic layers,^38−40^ little is known about anisotropy in carrier diffusion inside the inorganic layers^41^ and its direct relation with electron–phonon interactions.

In this work, we systematically study the anisotropy of electron transport within the inorganic lamellae, and the contribution from coupling with specific phonons, as a function of confinement. Using large-area single crystals, we identify the crystallographic direction of the transition dipole moments (TDMs) of all optically active phonon modes in BAPI and observe the time/frequency-resolved photoconductivity along, and perpendicular to, the direction of the 1 THz phonon TDM (i.e., the [102] and [-201] directions). We observe a clear anisotropy in the photoconductivity, where the mobility is ∼5–10% larger/smaller in perpendicular/parallel direction. Combining density functional theory (DFT) simulations and Feynman polaron theory, we unveil that the apparent anisotropy in photoconductivity originates from anisotropic electron-vibration interactions with the 1 THz phonon mode.

We used a synthesis protocol adapted from literature^42^ (see section SI1 in the SI) to grow BAPI single crystals with a large lateral area (>1 cm^2^) and high *n* purity, sufficiently thin (∼10–200 μm) to transmit photons in the THz frequency range. Large-area single crystals are a prerequisite for probing anisotropy through THz spectroscopy due to the diffraction-limited size of our THz pulse (∼1 mm diameter). Figure 1(a) shows photographs of representative crystals at the liquid–air interface. BAPI crystals consist of layered, primarily inorganic sheets, electronically decoupled by BA. By changing the ratio of BA and MA ions, we control and vary the thickness of the inorganic structure between *n* = 1–4. The bottom part of the figure shows the extended unit cells of the BAPI crystals, where we have used the convention to denote directions ⟨*h*0l⟩ in the inorganic planes of the structure. The high phase purity of the BAPI crystals is shown both by the powder X-ray diffraction (pXRD) measurements in Figure 1(b) as well as the reflectivity spectra, shown in Figure 1(c). From the pXRD, we determine the interlamellar spacing, *d*_010_, to increase from 13.77 ± 0.04 Å for *n* = 1 to 32.17 ± 0.05 Å for *n* = 4, an increase of 6.1 ± 0.2 Å/*n*, i.e., precisely the size of one methylammonium lead iodide octahedron (see SI section SI2). The different crystals all adopt an orthorhombic crystal structure, albeit with different space groups: *Pbca* for n = 1^21^, and *Cc2m*, *C2cb* and *Cc2m* for *n* = 2–4 respectively.^11^ The reflectivity spectra in Figure 1(c) for all different layer thicknesses show two features: a transition at higher energy, which corresponds to the excitonic absorption line, and a sub-bandgap feature at longer wavelengths,^43−46^ both of which are indicated by vertical dashed lines in the spectra.

**Figure 1:**
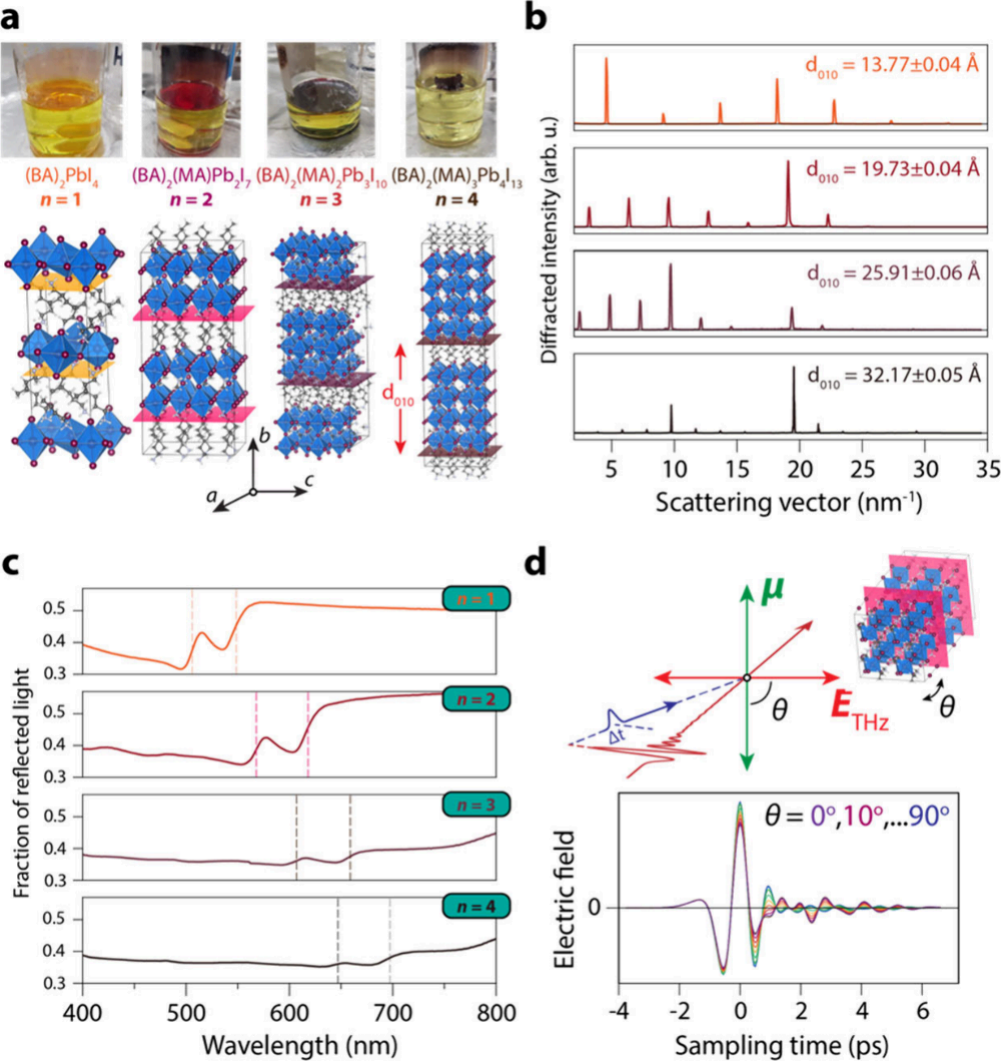
Structure and linear optical properties of two-dimensional single-crystalline BAPI and schematics of the polarization-resolved ultrafast THz spectroscopy. (a) Top row: photographs of single-crystalline (BA)_2_PbI_4_ (*n* = 1), (BA)_2_(MA)Pb_2_I_7_ (*n* = 2), (BA)_2_(MA)_2_Pb_3_I_10_ (*n* = 3) and (BA)_2_(MA)_3_Pb_4_I_13_ (*n* = 4). Bottom row: extended unit cells of 2D BAPI. (b) Powder XRD of single-crystalline BAPI sheets with different *n*, revealing the lamellar structure and the high “*n*-purity” of the crystals. (c) Reflectance spectra for the 2D BAPI crystals with different *n*, showing the excitonic and subgap resonances. (d) Schematic of the polarization-resolved linear THz time-domain spectroscopy (TDS) and optical-pump/THz-probe (OPTP) experiments. As we rotate the crystal, the relative orientation of the THz electric field vector, **E**_THz_, with respect to transition dipole moments μ of vibrational-modes, changes. When **E**_THz_ || μ, phonons can absorb the THz field; when perpendicular, they cannot. The bottom panel shows a transmitted THz pulse through an *n* = 1 BAPI single crystal, as a function of the azimuthal angle, θ, of the crystal. In OPTP experiments, the THz field will accelerate photoexcited carriers along its electric field polarization, probing the mobility along that axis.

Figure 1(d) shows a schematic of the polarized THz (pump–probe) spectroscopic experiments. A linearly polarized THz pulse (with frequencies between 0.3–3 THz), impinges on the BAPI crystals along the surface normal of the inorganic lamellae. By rotating our sample, we effectively align the TDMs (**μ**) in the plane of the crystal w.r.t. the electric field vector of our THz photons (with θ the azimuthal angle between **E**_THz_ and **μ**). When **E**_THz_ || **μ** (θ = 0°), the field couples to this TDM and gets attenuated; when they are perpendicular (θ = 90°), they cannot interact and the field is simply transmitted. We also performed optical-pump/THz-probe (OPTP) measurements, where we photoexcite our samples at 400 nm, and probe the optical conductivity via the change in the transmitted THz electric field,^47^ -Δ*E*/E_0_, with Δ*E* the difference in transmission between the photoexcited and unexcited BAPI crystal, and E_0_ the steady-state transmittance of the THz pulse. Here, the charge carriers are accelerated by the THz field in a specific crystallographic direction in the crystals, given by θ. We measured the OPTP transients parallel and perpendicular to the 1 THz TDM, as the modes at this frequency have been shown to dominate electron–phonon interaction.^26−28,48^

Figure 2(a-d) show the absorption spectra in the 0.3–3 THz frequency range. For *n* = 1 BAPI, shown in Figure 2(a), there are four distinct modes. Compared to the THz absorption spectrum of bulk MAPI, which has two modes at 0.9 THz (octahedral rocking mode) and 2 THz (Pb–I–Pb stretch),^25,49^ it seems the modes are split. This can be rationalized due to the lower symmetry of the *n* = 1 BAPI crystal; the in-plane and out-of-plane vibrations in BAPI sample different potential energy surfaces compared to bulk MAPI. Indeed, below the tetragonal-to-orthorhombic phase transition temperature, the two vibrational modes of bulk MAPI split into four, a result of the reduced crystal symmetry, lifting the degeneracy of the modes.^50^ Furthermore, the absorption of the modes at 0.9, 1.2, and 2.5 THz can be switched on and off by rotation over θ, the difference in optical density being the polarization contrast, where the former two and the latter are out of phase (i.e., they have perpendicular TDMs). The mode at 2 THz shows no dependence on θ, which can be the case if there is a high apparent degeneracy. Indeed, according to the theory presented below, this peak in the absorption spectrum comprises many different eigenmodes. As these eigenmodes all have different directionalities of their TDMs, this mode can be seen as quasi-degenerate.

**Figure 2:**
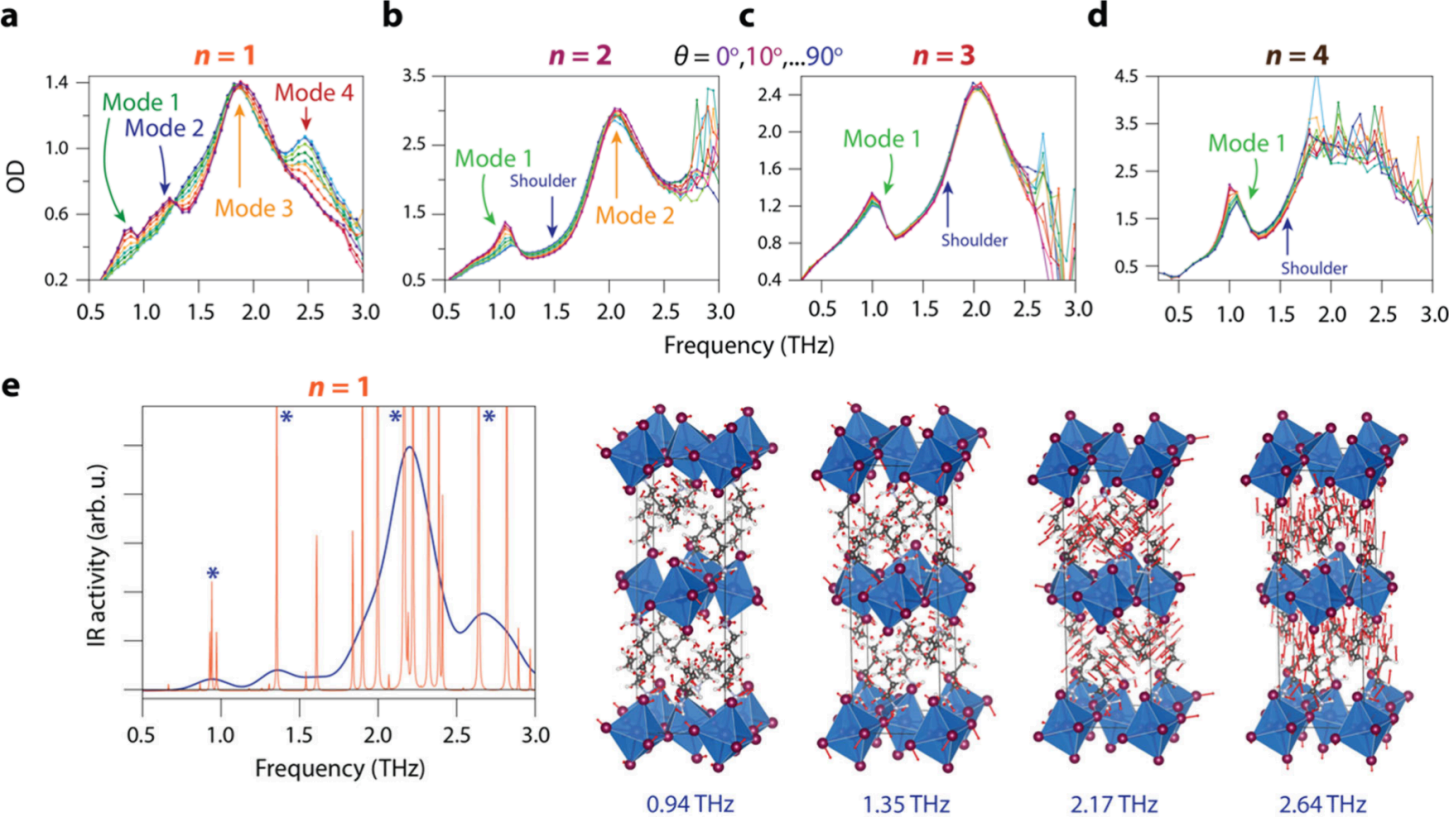
THz transmission measurements through the single-crystalline BAPI crystals as a function of azimuthal angle and simulated eigenmodes for *n*= 1 BAPI. (a) Extinction spectra for *n* = 1 BAPI as a function of θ. Four different modes are identified. (b) Extinction spectra for *n* = 2 BAPI as a function of θ. (c) Extinction spectra for *n* = 3 BAPI as a function of θ. (d) Extinction spectra for *n* = 4 BAPI as a function of θ. Notice how, with increasing *n*, the θ-dependence and polarization contrast of the modes decreases. (e) Simulated eigenmodes for *n* = 1 and the corresponding absorption spectrum. The atomic displacement vectors of the four modes with the highest oscillator strength, indicated with a (*) in the spectrum, are displayed on the right.

When increasing *n* in Figures 2(b-d), the peak splitting we observed in *n* = 1 is reduced. For *n* = 2, we observe that the mode at 1.1 THz has a θ-dependent region at the lower frequency side of the mode, and a θ-independent part at the high frequency side of the mode. The “on–off ratio” of this 1 THz mode is strongly reduced compared to *n* = 1, and decreases further for *n* = 3 and 4. For all *n*, the intense mode around 2 THz shows no θ-dependence. Note that for *n* = 4, we could not synthesize sufficiently thin single crystals to achieve reasonable transmission of THz photons with frequencies above 1.6 THz.

To understand the drastic change in the angle-dependent behavior of the vibrational spectra observed when changing the layer thickness microscopically, we computed the THz absorption spectra for *n* = 1 from DFT-based harmonic phonon theory (computational details^51−60^ can be found in the SI). We separately compute spectra for the high-temperature (HT) and low-temperature (LT) phases (*T*_c_ ≈ 274 K) of n = 1 BAPI based on resolved crystal structures from ref. [19]^21^, (see SI Figure S10). Surprisingly, the calculated spectrum of the LT phase matches the room temperature, i.e. above *T*_c_, experimental data well. We believe this is due to the proposed disordered nature of the HT phase,^21^ i.e., that the HT phase locally resembles the LT phase, and we thus choose to use the LT structural model in the following analysis. Figure 2(e) shows the calculated IR spectrum for the LT phase convoluted with a 0.1 THz Gaussian broadening (blue line) and when a small broadening is applied to each phonon mode (orange line). The overall shape of the broadened spectrum, barring a slight blue shift, agrees well with our experimental measurements in Figure 2(a). There are four clear peaks with obvious correspondence to the four peaks of the measured extinction spectrum. It thus becomes clear that each of these peaks, in fact, contains various numbers of eigenmodes that all have significant TDMs. The eigendisplacements of the phonon modes with the strongest TDMs for each peak, indicated with an asterisk in the calculated spectrum, are shown on the right-hand side of Figure 2(e). These displacements are made up of various distortions of the PbI_6_ octahedra, but also significant translational motion of the butylammonium cations, especially for the two higher frequency modes.

Figure 3(a-d) shows the angle-dependence of the observable modes extracted from the data in Figure 2, correlated to the crystallographic orientation of the crystals, which we measured by performing transmission XRD on the same crystal we performed the THz experiments on. For all the thicknesses, the TDM of the 1 THz modes lies in the ⟨201⟩ and ⟨102⟩ family of directions, which differ only slightly due to variations in the unit-vector lengths in the *a* and *c* direction of the crystals. The mode at 2.5 THz is perpendicular to this direction in *n* = 1 and lies in the ⟨1̅02⟩ direction.

**Figure 3:**
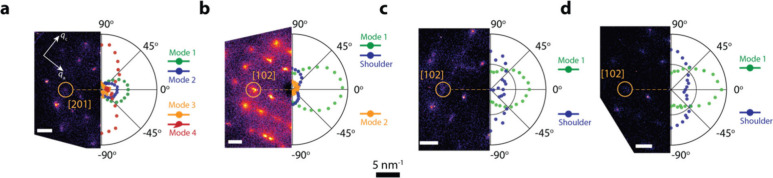
Transition dipole moment vectors of the vibrational modes in 2D BAPI. (a) Single-crystal diffraction pattern of the *n* = 1 BAPI crystal (left) and the intensity of the vibrational modes (right). The transition dipole moment (TDM) of the two modes around 1 THz lies in the [201] crystallographic direction, the TDM of the 2.5 THz mode is orthogonal to the former and lies in the [1̅02] direction. (b) Single-crystal diffraction pattern of the *n* = 2 BAPI crystal (left) and the intensity of the vibrational modes (right). The TDM of the vibrational mode around 1 THz lies in the [102] crystallographic direction. (c) Single-crystal diffraction pattern of the *n* = 3 BAPI crystal (left) and the intensity of the vibrational modes (right). The TDM of the vibrational mode around 1 THz lies in the [102] crystallographic direction. (d) Single-crystal diffraction pattern of the *n* = 4 BAPI crystal (left) and the intensity of the vibrational modes (right). The TDM of the vibrational mode around 1 THz lies in the [102] crystallographic direction. For all different *n*, the intense mode around 2 THz shows no angle dependence.

We have also calculated an angle-resolved IR-spectrum from our phonon simulations as shown in the SI (section SI6). We note that, since we employ the harmonic phonon approximation, the symmetry of the structure implies that the TDMs of all modes lie within the ⟨100⟩, ⟨010⟩ or ⟨001⟩ family of directions. This does not match our experimental observation, and it is thus likely that higher-order effects, e.g. anharmonic phonon–phonon coupling, result in overall TDM in the off-diagonal ⟨102⟩ directions.

Next, we compare the complex photoconductivity between directions parallel and perpendicular to the directions of the 1 THz TDMs.^61^Figure 4(a-d) shows the real and imaginary parts of the OPTP transients along both directions after photoexcitation at 400 nm. Noticeably, the transient amplitudes exhibit anisotropy persistently for all the measured thicknesses (*n* = 1–4) over the experimental time window (∼20 ps). We note that all the OPTP measurements are performed in the low-fluence, i.e. linear, regime, to exclude the presence of higher-order recombination processes originating from carrier–carrier interactions, and to prevent photodegradation of the crystals. Furthermore, for each *n*, we kept the same excitation fluence (within 1%) for measurements parallel and perpendicular to the 1 THz TDM.

To dissect the overall carrier dynamics, conduction mechanism and their anisotropies, we first consider the decaying components for each thickness, *n*. For *n* = 1–3, shown in Figure 4(a-c), the OPTP signal shows a fast decay over the first couple of picoseconds, followed by slower decay at later times. Comparing the resonant excitation of the crystals (Figure S13) with excitation at 400 nm (Figure 4(a-c)), we interpret that the initial decay upon 400 nm excitation comes from highly mobile hot carriers, which cool within a few ps to less mobile states at the band edges, an effect previously observed in MAPI^62^ and for hot holes in the Cs_2_AgBiBr_6_ double perovskite.^63^ As the layer thickness increases, we expect the decay of the photoconductivity to become second-order-like, as in MAPI, where the photoconductivity does not decay over the first nanoseconds.^64,65^

Next, we examined the in-plane photoconduction mechanism by recording photoconductivity spectra at a pump–probe delay time of 10 ps. We numerically retrieved the refractive index from an analytical model of the transmission of the THz pulse through the sample^66−68^ (see SI section SI5). The conductivity spectra along both directions for each thickness, are shown in Figure 4(e-h). Surprisingly, for all samples, we obtain a conductivity spectrum that does not resemble an excitonic response in our frequency range (a negative imaginary- and zero real photoconductivity, i.e., a Lorentzian oscillator for an interexcitonic transition) at 10 ps pump–probe delay time. Instead, the positive real, and almost zero imaginary photoconductivity for all *n* looks Drude-like. Indeed, we can fit our data with the Drude model, from which we obtain the plasma frequency (proportional to the carrier density) and the carrier scattering time.

**Figure 4:**
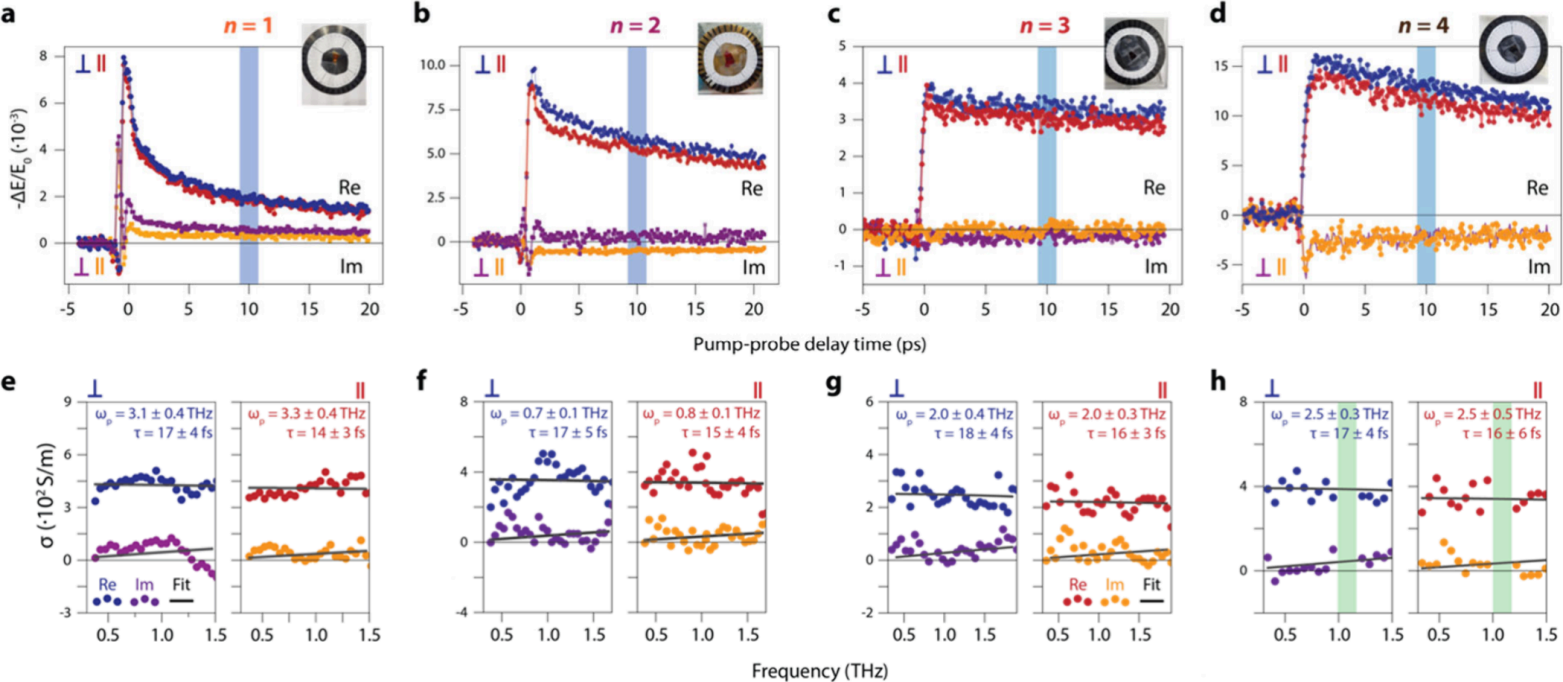
Polarization-resolved optical-pump/THz-probe (OPTP) experiments on the 2D BAPI single crystals. All samples are excited at 400 nm at low fluence (i.e., in the linear regime) and additional care was taken for each *n* to measure the parallel and perpendicular photoconductivity at the same pump fluence. (a) Real and imaginary OPTP traces for *n* = 1. (b) Real and imaginary OPTP traces for *n* = 2. (c) Real and imaginary OPTP traces for *n* = 3. (d) Real and imaginary OPTP traces for *n* = 4. The blue vertical bars in (a–d) indicate at which pump–probe delay time we acquired conductivity spectra and averaged the OPTP signal. (e–h) Conductivity spectra for different *n* measured at a pump–probe delay time of 10 ps. Left and right panels show conductivity spectra obtained with the THz polarization perpendicular and parallel to the 1 THz transition dipole moments, respectively. Solid lines are fits to the Drude model. In panel (h), the vertical green bars indicate the spectral range in which the transmittance of the THz field was lower than 2%, which was omitted from further analyses.

The results of all the photoconductivity experiments are summarized in Figure 5. We fitted the OPTP transients with a model that contains the sum of two decaying exponentials convolved with the IRF (except for *n* = 4, where we only needed one single decaying exponential to fit our data), of which the fitted decay rates are shown in Figure 5(a). These decay rates show little to no dependence on the direction in which we probe the photoconductivity.

The scattering times vs the inorganic layer thickness *n* are obtained from fitting the Drude model to the conductivity spectra in Figure 4(e-h), and are shown in Figure 5(b). For all the different layer thicknesses, these scattering times are quite similar, around 17 fs, however we observe that in all samples, the scattering time in the direction perpendicular to the 1 THz TDM (i.e., the ⟨102⟩ direction) is consistently higher than in the direction parallel to it (i.e., the ⟨2̅01⟩ direction), albeit within each other’s standard deviation. We compare the photogenerated carrier quantum yields, defined as the carrier density obtained from the plasma frequencies divided by photogenerated carrier density, in Figure 5(c). Both the plasma frequencies and the quantum yields do not show anisotropy vs *n*, indicating that in both directions, the generated carrier densities are identical and excluding this as a cause for the apparent anisotropy in photoconductivity. The value of the quantum yield, around 30%, is in line with earlier observations in 3D perovskites.^5,69^

We quantified the anisotropy in the photoconductivity in Figure 5(d), where we show the average of the real photoconductivity from the OPTP traces around 10 ps [shown by the vertical blue bars in Figure 4(a-d)], the average of the real part of the photoconductivity spectrum [shown in Figure 4(e-f)] and the scattering times obtained from the Drude fits as a function of *n* and for both perpendicular and parallel directions to the 1 THz TDM (see SI, section SI7). We defined the anisotropy ratio as the value perpendicular to the 1 THz TDM divided by the value parallel to the 1 THz TDM. For all different *n*, the apparent photoconductivity is higher in the direction perpendicular to the 1 THz TDM, compared to parallel to it. The estimated anisotropy of the photoconductivity is ∼10% for all samples.

To explain these findings, we turn to the Feynman variational polaron solution of the extended Fröhlich polaron model Hamiltonian. The original Feynman theory explicitly includes multiple phonon modes^70^ and anisotropic effective masses.^71^ Here, we use the calculated anisotropic modes in the LT phase, associate a Fröhlich dielectric mediated electron–phonon coupling with the infrared activity of each mode, and solve the finite temperature mobility theory for 300 K. We find a Fröhlich dimensionless electron–phonon coupling of 3.44 and 3.75 along the two in-plane lattice vectors; both considerable higher than in 3D halide perovskites. This leads to predicted mobilities of 1.89 cm2/V/s and 1.76 cm2/V/s in the two directions, an anisotropy of 8%, and a value close to the experimentally obtained anisotropy estimates. The additional electron–phonon coupling also increases polaron localization, by a similar quantity. We, therefore, explain the experimentally observed anisotropy in photoconductivity to be due to different intrinsic carrier mobilities, arising directly from the anisotropy in the dielectrically mediated electron–phonon coupling strength. Overall, the impact of the 1 THz phonon mode on perovskite electronic properties is remarkable.^27^

To summarize, we have studied anisotropy in the phonon transition dipole moments and the optical conductivity of single crystalline two-dimensional BAPI perovskites. Through a unique combination of synthesis, characterization and analysis, we unravelled the crucial role of electron–phonon interactions on directional carrier transport in these perovskite materials. From the linear THz absorption measurements, we determine the TDM vectors of the 1 THz modes to lie in the ⟨102⟩/⟨201⟩ family of directions and show their evolution with different thicknesses of the inorganic layer *n*. Furthermore, optical-pump/THz probe spectroscopy experiments show that the photoconductivity is 5–10% higher in the direction perpendicular to the 1 THz TDM compared to the direction parallel to it. Theoretical calculations based on Feynman’s polaron theory corroborate the observed anisotropy and pinpoint that directional electron–phonon interactions are likely responsible for this effect. Our results shed new light on some of the fundamental molecular physics governing direction-dependent effects in these quasi-two-dimensional perovskite semiconductors and corroborate the importance of the dynamic interplay between vibrational modes and charge carrier motion in these materials.

**Figure 5:**
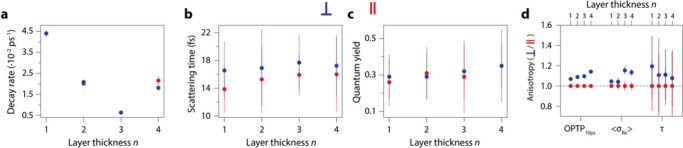
Results from fitting the Drude model to the conductivity spectra and hints of anisotropy in the photoconductivity in 2D single crystalline BAPI. In all panels the blue data points are data obtained with a THz polarization perpendicular to the 1 THz transition dipole moments, and the red data points are obtained parallel to the 1 THz TDM. (a) Slowest decay rate vs layer thickness, obtained from fitting the real part of the OPTP traces [Figure 3(a-d)]. (b) Scattering times vs *n*. (c) Quantum yield vs *n*, which was obtained by comparing the absorbed photon density to the carrier density calculated from the plasma frequency. (d) Averaged real part of the OPTP signal [blue vertical bars in Figure 3(a-d)], averaged real part of the conductivity spectra, and scattering times at 10 ps pump–probe delay time vs *n*. Note how all the blue data points are higher for all *n* than the red data points, indicating that the photoconductivity in 2D BAPI is ∼10% higher in the direction perpendicular to the 1 THz TDM compared to parallel to it.

## Supporting Information Available

Detailed description of the interfacial synthesis method, description of experimental techniques, data analysis of the THz TDS and OPTP experiments, description of simulation methods, Tables S1–S4, and Figures S1–S14 (PDF)
