# Supplementary material for: Anisotropic Electron–Phonon Interactions in 2D Lead-Halide Perovskites
Source: Nano Lett. 2024 Jul 8;24(28):8642–9. doi: 10.1021/acs.nanolett.4c01905 (PMC11261630; doi:10.1021/acs.nanolett.4c01905)
Supplement: Supplementary file 1 — nl4c01905_si_001.pdf [file nl4c01905_si_001.pdf]

Supporting information

Anisotropic electron-phonon interactions in 2D lead-halide perovskites

Jaco J. Geuchies[1*], Johan Klarbring[2,3], Lucia Di Virgilio[1], Shuai Fu[1], Sheng Qu[1], Guangyu Liu[4], Hai Wang[1], Jarvist M. Frost[4], Aron Walsh[2], Mischa Bonn[1*] and Heejae Kim[1,5*]

1. Max Planck Institute for Polymer Research, 55128 Mainz, Germany.

2. Department of Materials, Imperial College London, London SW7 2AZ, United Kingdom.

3. Department of Physics, Chemistry and Biology (IFM), Linköping University, SE-581 83, Linköping, Sweden

4. Department of Physics, Imperial College London, London SW7 2AZ, United Kingdom.

5. Department of Physics, Pohang University of Science and Technology, 37673, Pohang, Korea.

*Corresponding authors

Table of Contents

SI1 - Materials and methods **3**

SI2 - Additional steady-state characterization **7**

SI3 - Optical-pump/THz-probe spectroscopy **9**

SI4 - Analytical model for the transmittance of THz pulses and photoconductivity through the quasi-2D perovskite single crystals **12**

SI5 - Numerical minimization to obtain complex refractive indices and photoconductivity in the ground- and photoexcited state **14**

SI6 - Computational details **20**

Phonon band structures and IR spectrum 22

Directionally resolved IR spectrum 23

Electronic band structure 24

Electron-phonon interaction and directional photoconductivity 24

SI7 – Anisotropy ratio determination from THz spectroscopy **27**

SI8 - Supplementary references **28**

SI1 - Materials and methods

Chemicals PbO (>99.9%), hydroiodic acid (57% w/w in water), hypophosphorous acid (H_3PO_2, 50% w/w in water), n-butylamine (99.5%) were purchased from Sigma-Aldrich. CH3NH3I was obtained fromLuminescence Technology Corp. Methylammonium chloride (low water content) was bought from TCI. All chemicals were used without further purification.

Synthesis procedure. The synthesis of cm-sized crystal flakes was done at a liquid-air interface, based on an adjusted protocol by Wang *et al.*[1]. For the exact amounts, see table S1 below. We mixed PbO in HI (57% w/w solution in water) and H_3PO_2 (50% w/w solution in water) in a 20mL glass vial. The mixture was heated to 80°C, at which point the PbO dissolved and a clear yellow solution was obtained. The thermocouple that controls the hot-plate temperature was always inserted in a 20 mL glass vial filled with the same volume of water as the perovskite precursor solutions.

Next to this, a solution of n-butylamine (nBAm) and methylammonium (MA) chloride in HI was prepared while cooling the vial, holding the nBAm and MA, in an icebath. Care should be taken to add the HI slowly, as the reaction is very exothermic (one can observe fumes forming as the HI is added). This nBAm/MA solution is dropwise added to the Pb-containing solution. Upon mixing the two solutions, some small crystallites are formed, which dissolve rapidly.

Depending on the layer thickness that is formed, crystal growth is done either by lowering the temperature from 80°C to 70°C for n=1 and n=2, and crystal growth takes 10-30 minutes. For n=3 and n=4, we used a temperature controller (JKEM, controller type T), with a glass-coated probe inserted in a vial filled with water on the same hot plate, and ramped the temperature

down by 2°C per hour until nucleation of a crystal at the liquid-air interface, at which point the temperature was kept constant (usually between 55-65°C).

After the growth of the crystals at the liquid-air interface, the crystal is scooped gently off the interface with Teflon tweezers, placed on a plastic lid, and dried in a vacuum oven for 5 hours. It is important to attach a liquid N2 cold-trap in between the oven and the vacuum pump, to catch off the remaining water-HI solution. After drying, the samples are stored in a nitrogen-filled glovebox until further use. Note that the overall crystal thickness varies from 10-20 micrometers for *n*=1 (making them very fragile) up to 200 micrometers for *n*=4.

Additionally, for the layers where $n > 1$, we used MACl (instead of MAI). For reasons unclear to us, we were able to get interfacial nucleation more consistently (instead of nucleation in the bulk of the liquid), using MACl compared to MAI.

Precursor quantities for the synthesis of $(BA)_2(MA)_{n-1}Pb_nI_{3n+1}$ single crystals

Table S1: amount of precursors used in the synthesis of the $(BA)_2PbI_4$ crystals (n=1)

n=1	Chemical	Amount (mmol)	Amount (mg)	Amount (mL)
**PbI solution**	PbO powder	5	1116	
	57% w/w acqueous HI solution	38		5
	50% (w/w I think) acqueous H_3PO_2 solution	7.75		0.85
**n-$CH_3(CH_2)_3NH_3I$ solution**	n-$CH_3(CH_2)_3NH_2$ (liquid)	5		0.462
	57% w/w acqueous HI solution	19		2.5

Table S2: amount of precursors used in the synthesis of the $(BA)_2(MA)Pb_2I_7$ crystals (n=2)

n=2	Chemical	Amount (mmol)	Amount (mg)	Amount (mL)
**PbI solution**	PbO powder	5	1116	
	57% w/w acqueous HI solution	38		5
	50% (w/w I think) acqueous H_3PO_2 solution	7.75		0.85
**n-$CH_3(CH_2)_3NH_3I$ solution**	CH_3NH_3Cl	2.5	169	
	n-$CH_3(CH_2)_3NH_2$ (liquid)	7		0.694
	57% w/w acqueous HI solution	19		2.5

Table S3: amount of precursors used in the synthesis of the $(BA)_2(MA)_2Pb_3I_{10}$ crystals (n=3)

n=3	Chemical	Amount (mmol)	Amount (mg)	Amount (mL)
**PbI solution**	PbO powder	5	1116	
	57% w/w acqueous HI solution	38		5
	50% (w/w I think) acqueous H_3PO_2 solution	7.75		0.85
**n-$CH_3(CH_2)_3NH_3I$ solution**	CH_3NH_3Cl	3.33	225	
	n-$CH_3(CH_2)_3NH_2$ (liquid)	1.67		0.164
	57% w/w acqueous HI solution	19		2.5

Table S4: amount of precursors used in the synthesis of the $(BA)_2(MA)_3Pb_4I_{13}$ crystals (n=4)

n=4	Chemical	Amount (mmol)	Amount (mg)	Amount (mL)
PbI solution	PbO powder	5	1116	
	57% w/w acqueous HI solution	38		5
	50% (w/w I think) acqueous H_3PO_2 solution	7.75		0.85
n-$CH_3(CH_2)_3NH_3I$ solution	CH_3NH_3Cl	3.75	253.5	
	n-$CH_3(CH_2)_3NH_2$ (liquid)	1.25		0.124
	57% w/w acqueous HI solution	19		2.5

SI2 - Additional steady-state characterization

X-ray diffraction was performed on a Rigaku Smartlab diffractometer, using copper Kα radiation (1.54 angstrom), both in Bragg-Brentano geometry for powder-XRD and in transmission geometry. From the powder XRD patterns, we fitted the peak position of the reflections. Since we are only sensitive to specular reflections in the out-of-plane directions in this geometry (i.e. reflections from the inorganic lamella), we could determine the interlayer spacing for each of the different thicknesses of BAPI.

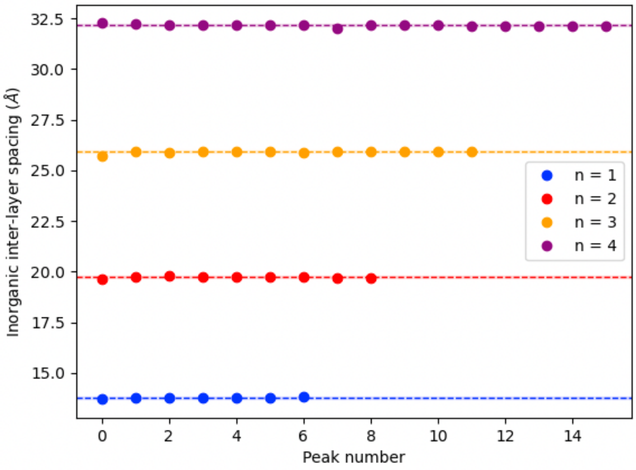

Figure S1: from the fitted p-XRD peak positions, we calculate the interlayer spacing, as shown and disussed in the main text.

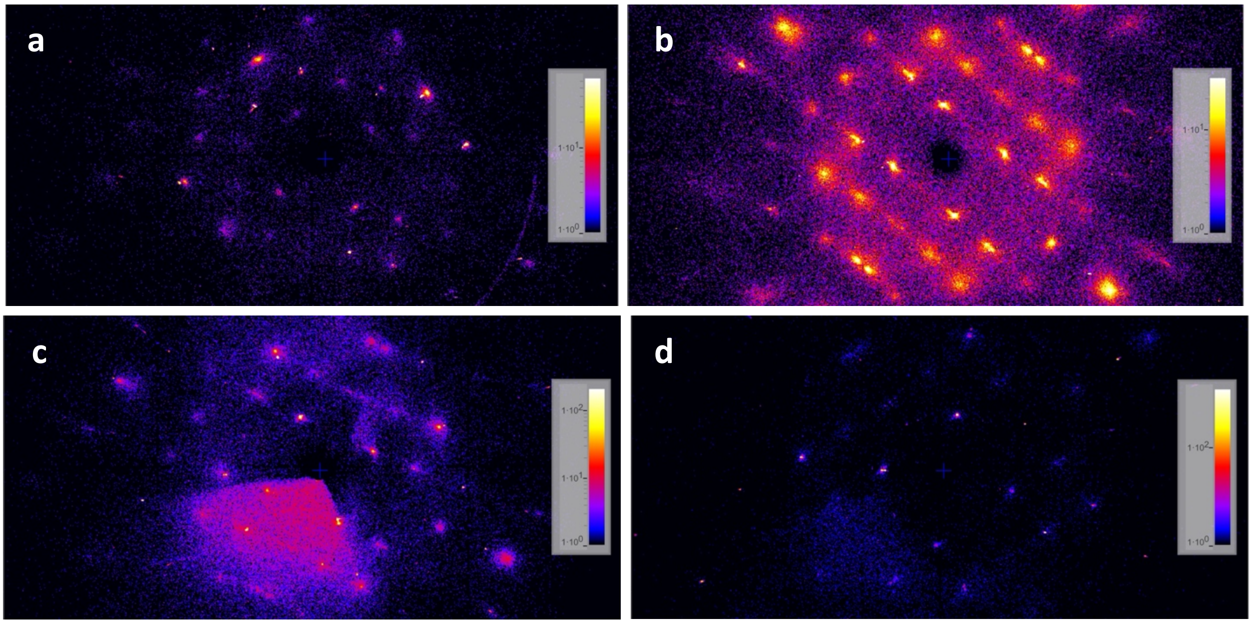

Figure S2: transmission 2D WAXS patterns from the samples shown throughout the main text. (a) n = 1 BAPI. **(b)** n = 2 BAPI. **(c)** n = 3 BAPI. **(d)** n = 4 BAPI. The triangular shaped wedge in panel **(c)** originated from grease that was unfortunately present on the Kapton foil that holds the beamblock for the direct beam.

Optical reflectivity curves were measured with a Perkin-Elmer Lambda 900 UV/Vis/NIR spectrometer, where samples were placed at the direct end of an integrating sphere to collect all reflected light.

The thickness of the samples was measured using a Dektak profilometer.

SI3 - Optical-pump/THz-probe spectroscopy

Here, we describe the technique THz time-domain spectroscopy (TDS) and Optical Pump THz Probe Spectroscopy (OPTP). This technique is able to probe the conductivity of carriers and further retrieve the charge carrier mobility μ.

We use an amplified Ti:sapphire laser producing pulses with 800 nm central wavelength and ~50 fs pulse duration at 1 kHz repetition rate. The THz field is generated by optical rectification in a ZnTe(110) crystal (thickness 1 mm). The THz detection is based on the electro-optic (also called Pockels) effect in a second ZnTe crystal with 1 mm thickness. We vary the time delay between the THz field and the 800 nm sampling beam with a motorized delay stage (M-605.2DD purchased from Physik Instrument (PI)). The time delay between the optical pump and THz probe pulses is controlled by a second motorized delay stage (M521.DD, Physik Instrument (PI)). The pump pulse has a 400 nm central wavelength and is produced by the second harmonic generation of the 800 nm femtosecond pulse in a beta barium borate (BBO) crystal, where the remaining 800nm is filtered afterwards out with a short-pass filter (blue colorglass). For the excitation-wavelength dependent measurements, we used an optical parametric amplifier (OPA) to convert our 800nm fundamental beam into the various pump wavelengths, resonant with the band-edge excitonic transition of our materials, which we filter further using a bandpass filter for the corresponding wavelength directly after the OPA. The OPTP transients were measured at the peak of the THz waveform to measure the changed transmission with and without photoexcitation (proportional to the real part of the photoconductivity) and at the zero-crossing after the peak to measure the phase shift with and without photoexcitation (proportional to the imaginary part of the photoconductivity).

To determine the density of photoexcited carriers, we extracted the complex dielectric spectra from reference [61] of the main text. The injected photon density can be calculated as

$$n_{photon} = \frac{E\lambda}{hc} = \frac{Power\ (W)}{repetition\ rate\ (s^{-1}) \cdot \frac{h \cdot c}{\lambda}\ (J)\ \cdot \pi r^2\ (cm^2)}$$

Taking the reflectivity at the interface between two materials F_R into consideration, the absorbance of the perovskite film is

$$F_A \cdot F_R = (1) \cdot F_R = (1) \cdot \left|\frac{n_1 - n_2}{n_1 + n_2}\right|^2$$

where n_1 and n_2 are the refractive indexes of the materials, F_A is the fraction of absorbed light at the corresponding wavelength, which we set to 1 since the film thickness (>10 micron) is much larger than the absorption lengths for all BAPI forms. Here, $n_1 = 1$ (for air) and n_2 follows from reference [65] of the main text. The attenuation has been taken into consideration following the Beer-Lambert law, characterized by a decay length of a few tens nanometers from the surface, as

$$I(x) = I_0 e^{-\alpha x}$$

The corresponding absorbed light flux over the absorption length equals

$$\left(\frac{1 - e^{-\alpha L}}{\alpha L}\right) \cdot I_0$$

where α is the absorption coefficient (taken from the imaginary part of the complex refractive index κ as $\alpha = \frac{4\pi\kappa}{\lambda}$), and L is the absorption length (L = 1/α), again extracted from reference [65] in the main text. Considering all the above parameters, the modified absorbed photon density can be expressed as:

$$N_{abs} = \frac{Power}{repetition\ rate\ \cdot \frac{h \cdot c}{\lambda}\ \cdot \pi r^2\ \cdot L} \cdot \left|\frac{n_1 - n_2}{n_1 + n_2}\right|^2 \cdot \left(\frac{1 - e^{-\alpha L}}{\alpha L}\right) \cdot I_0$$

N is inferred from the absorbed carrier density, and is used to calculate the photon-to-carrier quantum yield Φ: $N = \Phi N_{abs}$, which is calculated from the fits to of the conductivity spectra to the Drude model, specifically from the plasma frequency:

$$N = \frac{\omega_p^2 m^* \varepsilon_0}{e^2}$$

with m* the reduced effective mass (of electrons and holes)[2], ε_0 the vacuum permittivity, and e the elementary charge.

The pump fluences used for the OPTP experiments were constant in between different single-crystals, but varied slightly between different n. We did make sure for all samples we measured in the linear regime. The fluences used to generate the data in figure 4 of the main text were 3.8±0.1 uJ/cm^2, 7.2±0.1 uJ/cm^2, 9.2±0.1 uJ/cm^2, and 20.2±0.1 uJ/cm^2 for n = 1, 2, 3 and 4 respectively.

Sample stability during pump-probe measurements: For the OPTP measurements, we verify we are in the low-fluence or linear regime. Furthermore, at higher fluences we observe sample degradation during the measurement, over the course of roughly two hours, which can be observed by eye by the loss of photoluminescence from the sample. After checking initial samples to find a suitable stability window in terms of fluence, the data presented throughout this paper is optimized such that we do not have loss of photoluminescence, which seemed to be a good indicator for sample stability. Furthermore, we do note that spincoated films of BAPI photodegrade much faster than the single crystals we show here.

SI4 - Analytical model for the transmittance of THz pulses and photoconductivity through the quasi-2D perovskite single crystals

We do *not* use the Tinkham approximation[3], i.e. the thin-film approximation, to calculate the photoconductivity spectra. The reason for this is that 1) the crystals are relatively thick (tens to hundreds of micrometers), and 2) the ground-state response in the THz frequency range is not flat due to the presence of vibrational resonances[4–7]. Instead, we write down the analytical Fresnel equations for the complex transmission of a THz field at a given frequency:

$$T_{calc}\,(f) = \frac{E_{sample}}{E_{free}} = \frac{t_{01}p_1t_{10}FP_{010}}{p_0} \qquad \text{(eq. S1)}$$

With T_{calc} the complex transmission at a given frequency *f*, $E_{sample/free}$ the amplitude of the THz electric field over a length *L* (the thickness of the crystal, measured with a profilometer) with the free-standing single crystal sample or without anything in its' path respectively. The transmission through the air-perovskite interface, t_{01}, propagation through the perovskite crystal, p_1, transmission through the perovskite-air interface, t_{10}, and propagation of the THz pulse through the sample or air over a length L are specified. Furthermore, due to the high refractive index of the perovskite material, Fabry-Perot-like multiple reflections are possible, captured by the term FP_{010}. These Fresnel coefficients are given by:

$$t_{01} = \frac{2}{1+n_C} \qquad t_{10} = \frac{2n_C}{1+n_C} \qquad P_1 = e^{\frac{i2\pi f L n_C}{c}} \qquad P_0 = e^{\frac{i2\pi f L}{c}}$$

$$r_{10} = r_{01} = \frac{n_C - 1}{n_C + 1} \qquad FP_{010} = \frac{1}{1 - r_{10}^2 p_1^2}$$

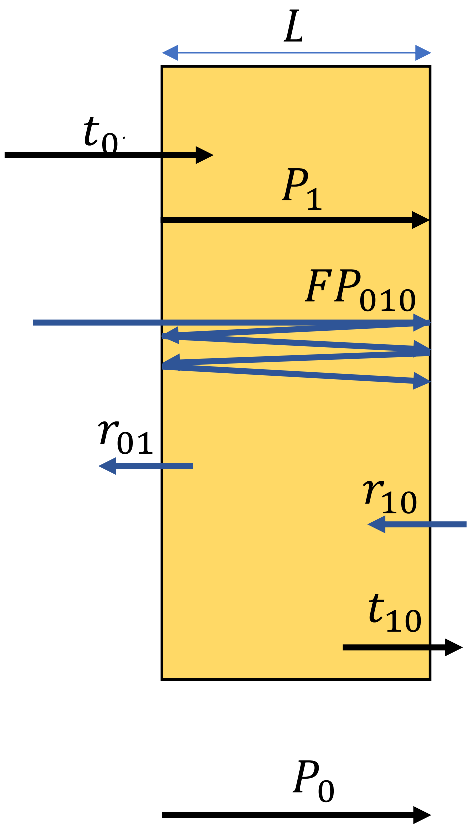

Figure S3: schematic of the transmission pathways of a photon at a given frequency through a sample of thickness *L*.

Here, in the Fabry-Perot term, we have taken the limit of an infinite number of internal reflections through the sample, which converges the geometric sum. We believe this is a valid approximation as each increasing term in this sum gets significantly smaller due to absorption through the material. We use equation S1 to numerically retrieve the refractive index of our perovskite single crystal in the THz frequency range, for all the different sample orientations we measured, in both the ground- and photoexcited state.

SI5 - Numerical minimization to obtain complex refractive indices and photoconductivity in the ground- and photoexcited state

Data pre-treatment: acquiring the proper phase. We now use the experimentally obtained complex transmission to obtain the real and imaginary parts of the complex refractive index. Since we measure the electric field of our THz pulse, we directly are able to measure both amplitude and phase, as shown below.

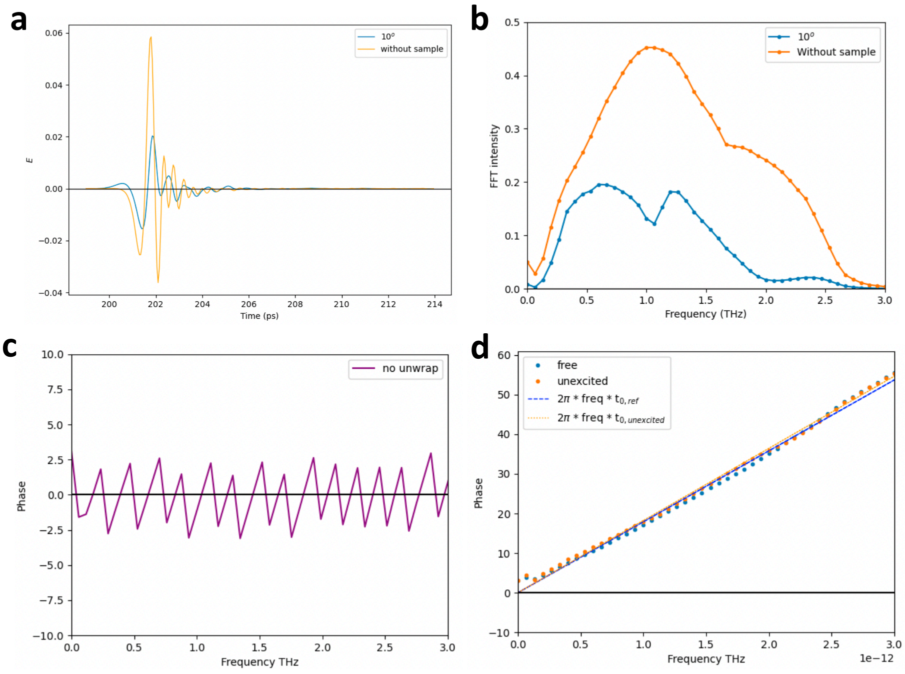

Figure S4: THz-TDS data and data analysis for n = 1. (a) Example of measured THz waveforms wit hand without the single-crystal in the THz beampath. **(b)** Absolute value FFT of the data shown in **(a)**, giving us the amplitude spectrum. Due to the relative thickness of the film, the vibrational modes are already clearly observed. **(c)** Raw, (incorrectly) unwrapped, phase of the THz fields, defined as the arctan(Imaginary/Real) for each frequency of the Fourier-transformed data from **(a)**. **(d)** Properly unwrapped phase as a function of frequency.

We obtain the experimental complex transmission as a function of frequency of our data, shown in Figure S4 above, as T_{sample} = FFT(E_{sample})/FFT(E_{free}). We can now rewrite our complex transmission with the properly unwrapped phase, which is important for the numerical minimization further ahead:

$$T_{sample} = |T_{sample}|e^{i\phi} \quad \text{(eq. S2)}$$

with ϕ being the unwrapped phase.

Numerical minimization. Since we now have the complex transmission of the sample, we can numerically minimize the mismatch of the calculated value (from equation S1) against our experimental data. To this end, we compute the difference between the calculated and measured amplitudes and phases, ΔT and $\Delta\phi$ respectively, and sum up the squared difference between experiment and calculation over all frequencies in our THz window:

$$difference = \sum_{frequencies} \Delta T^2 + \Delta\phi^2 \quad \text{(eq. S3)}$$

This difference can be numerically minimized against the complex refractive index of the sample n_c through various strategies. We opted to use a double minimization scheme, starting with a Basinhopping minimizer, implemented in the scipy.optimize library of Python. This minimizer starts at different initial guesses for the refractive index, for which we used the refractive index from the Tinkham approximation as an initial guess, and let the minimizer run for either a fixed or predetermined number of minimization steps, an example of which is shown in Figure S5 below. The advantage of the Basinhopping method, is that it searches a large parameter space for a global minimum.

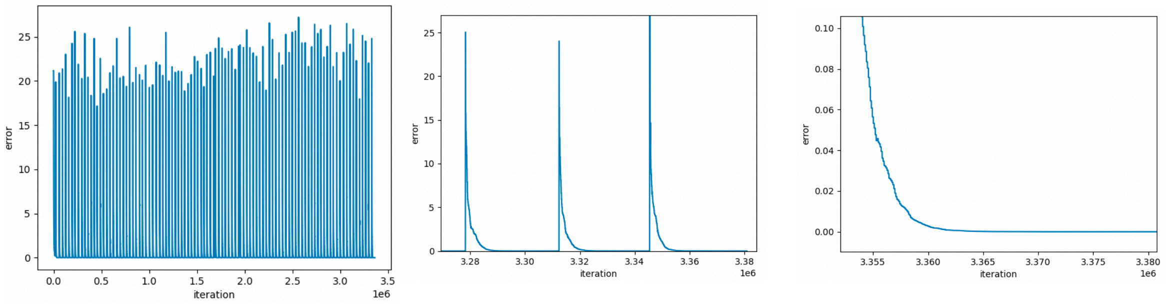

Figure S5: example of Basinhopping minimization, where from left to right, we zoom in more into the minimization steps.

The output of the Basinhopping algorithm does not check for convergence, so we run the minimized complex refractive index obtained through Basinhopping through a similar minimizer, scipy.minimize, using a Nelder-Mead algorithm to check for convergence.

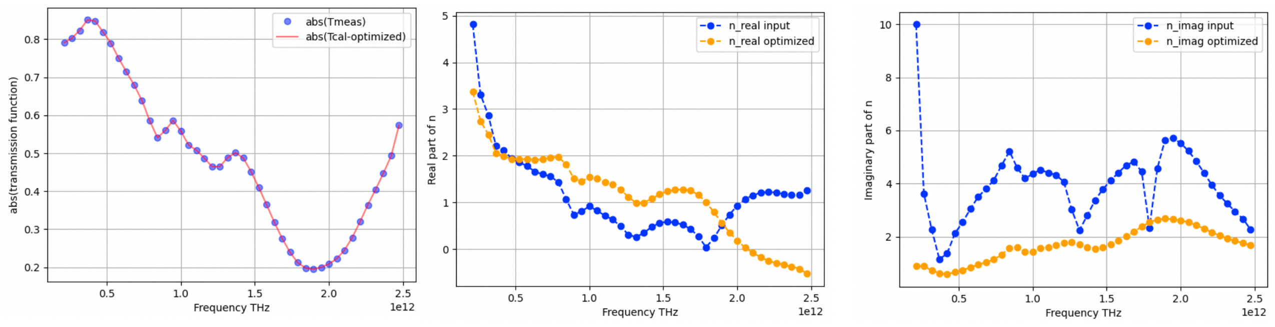

Figure S6: Example of minimized refractive index. The left panel shows the absolute transmission for both the experimental data, and through calculation with the minimized refractive index of the sample. The middle and right panels show the minimized real and imaginary parts of the refractive indices respectively, with in blue the initial guess and in yellow the minimized refractive index.

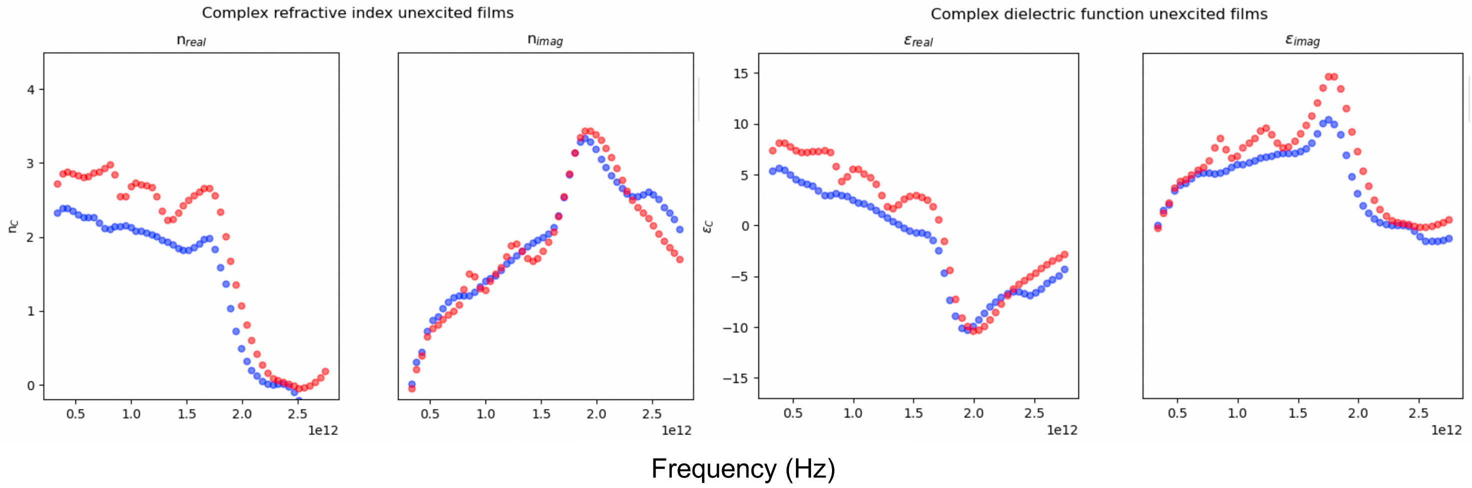

Figure S7: Imaginary and real parts of the refractive index (left) for the two extreme angles (90º apart) for n = 1 BAPI, which can be converted into complex dielectric functions (right).

We can apply the same minimization scheme to obtain the refractive index of the photoexcited sample. For this we calculate the electric field of the THz pulse transmitted through the photoexcited sample as

$$E_{photoexcited}(t) = E_{ground\ state}(t) + \Delta E(t) \quad \text{(eq. S4)}$$

Where we add the measured photoinduced change of the THz field, $\Delta E(t)$, to the transmitted field without photoexcitation $E_{ground\ state}(t)$. For the minimization, as an initial guess for the to-be optimized refractive index, we use the ground-state refractive index as an initial guess.

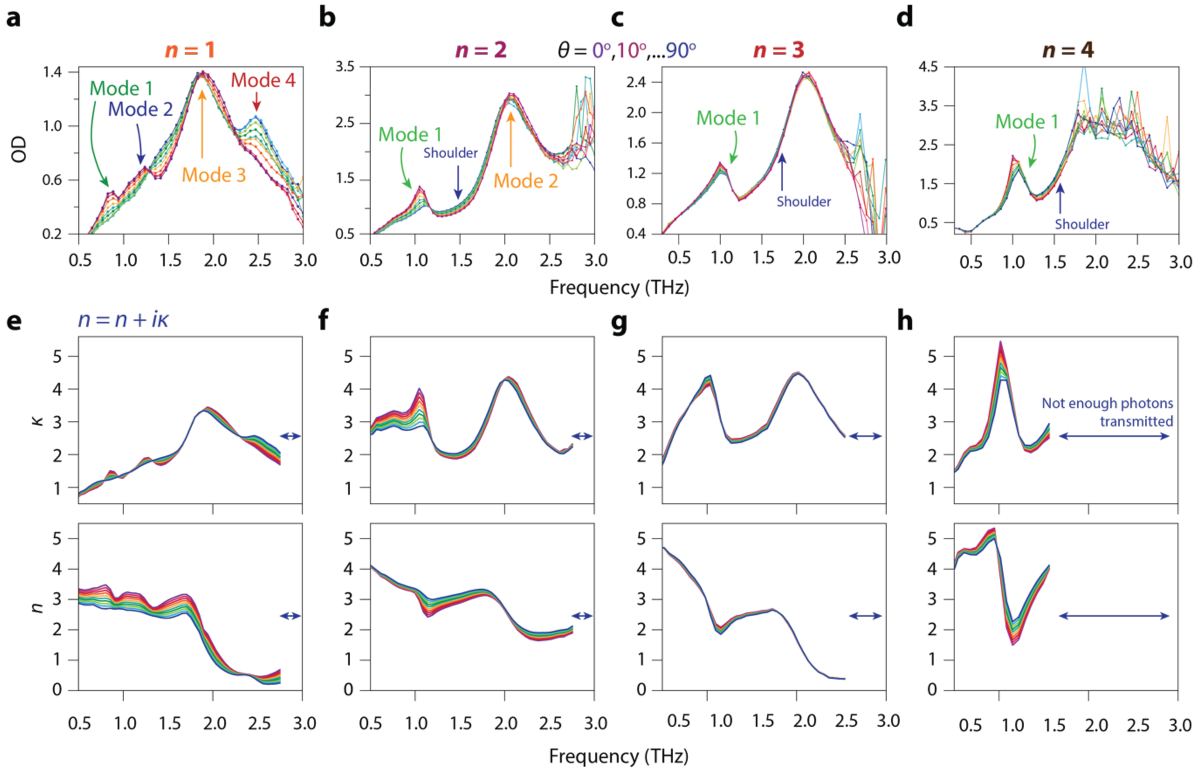

Figure S8: retrieved complex refractive indices in the THz spectral range for n = 1 – 4 BAPI. (a-d) optical density spectra for n = 1 to n = 4 going from left to right, as a function of angle. **(e-h)** Complex refractive indices for n = 1 to n = 4 BAPI going from left to right, as a function of angle. The top panels show the imaginary part of the refractive index, the bottom panels show the real part. The horizontal blue arrow indicates the spectral region that has less than 5% transmission of the THz field, which we omit from the numerical minimization due to the reduced signal-to-noise.

Obtaining the photoconductivity spectra. We convert the numerically retrieved refractive indices into dielectric functions, which are additive, and separate them into various terms.

$$n_c^2 = \varepsilon_c$$
$$\varepsilon_c = \varepsilon_{real} + i\varepsilon_{imag}$$

$$\varepsilon_{real} = n^2 - \kappa^2 \qquad \text{(eq. S5)}$$

$$\varepsilon_{imag} = 2n\kappa \quad \text{(eq. S6)}$$

Once we have the complex dielectric function of the sample ε_c, we decompose the total dielectric response of our system in the ground and photoexcited state as

$$\varepsilon_{c,groundstate} = \varepsilon_\infty + \varepsilon_{lattice} \quad \text{(eq. S7)}$$
$$\varepsilon_{c,excited\ state} = \varepsilon_\infty + \varepsilon_{lattice} + \varepsilon_{carriers} \quad \text{(eq. S8)}$$

Where we separate the total dielectric function into a constant contribution ε_∞, a lattice response $\varepsilon_{lattice}$ (i.e. vibrational modes that are present), and in the photoexcited state an additional response from the generated carriers $\varepsilon_{carriers}$. The latter term we want to isolate to obtain the conductivity of the photoexcited carriers: $\varepsilon_{carriers} = \varepsilon_{c,excited\ state} - \varepsilon_{c,groundstate}$. We convert this into a photoconductivity spectrum

$$\sigma(\omega) = -i\omega\varepsilon_0\varepsilon_{carriers}(\omega) \quad \text{(eq. S9)}$$

The photoconductivity spectra that we retrieved via this method are shown in Figure 4 of the main text and are fitted with the Drude model:

$$\sigma(\omega) = \frac{\omega_p^2\varepsilon_0\tau_s}{1-i\omega\tau_s} \quad \text{(eq. S10)}$$

With ω_p^2 the squared plasma frequency (proportional to the carrier density) and τ_s the Drude scattering time. This method has the advantage that the acquired photoconductivity spectra are analytical, and do not rely on various assumptions, e.g. the Tinkham/thin-film approximation, for the transmitted THz pulse.

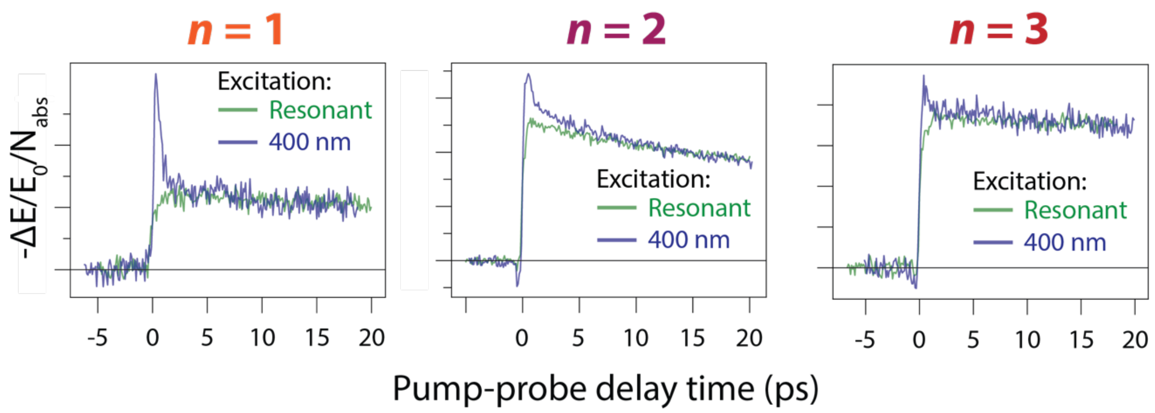

Figure S9: Hot carriers are more mobile than cold carriers – origin of the initial fast decay. OPTP traces for n = 1 (left), n = 2 (middle) and n = 3 (right) upon resonant band-edge excitation (green) and 400 nm excitation. Resonant excitation shows an IRF limited ingrowth of the OPTP transients, whereas 400 nm above-bandgap excitation shows a fast initial decay. Since the data is obtained at low photon fluences, i.e. in the linear regime, and normalized on the absorbed photon fluence, we can conclude that the initial decay originates from a higher mobility for hot carriers.

SI6 - Computational details

The density functional theory (DFT) calculations were performed using VASP[8–10] and the projector augmented wave (PAW)[11] methodology, with the PBE[12] exchange-correlation functional with added Tkatchenko-Scheffler[13] dispersion corrections (PBE+TS). For structural relaxation and phonon calculations, we used an 800 eV cutoff energy, a 6x6x2 Γ-centered k-point grid (where the simulation cell was aligned so that the c-direction corresponds to the out-of-plane direction), a convergence criterion for the electronic self-consistent iterations of 10^{-8} eV and a Gaussian smearing of the electronic states of 10 meV. Full structural relaxation was performed until the force on any atom was less than 0.5 meV/Å. For each element we used the VASP-recommended PBE PAW-potentials, labeled 'Pb_d', 'I', 'N', 'H', 'C', and evaluated

projectors in reciprocal space (LREAL = .FALSE.). The starting points for the structural relaxations of the n=1, LT and HT phases were taken from Ref. [14].

Harmonic phonon dispersion relations of the n=1, LT and HT phases were computed using the small-displacement formalism as implemented in Phonopy[15,16]. We used 2x2x1 supercells and a small-displacement distance of 0.01 Å.

Born effective charges (BECs) and dielectric tensors were calculated using VASPs density functional perturbation theory (DFPT) routines (LEPSILON = .TRUE.). Using the calculated BECs and Γ-point phonon frequencies and eigenvectors, IR spectra were obtained using the phonopy-spectroscopy package[17]; https://github.com/skelton-group/Phonopy-Spectroscopy]. We have cross-checked the small displacement phonon calculations for the LT phase with Γ-point eigenvectors and frequencies obtained directly from the VASP DFPT routines (LEPSILON = .TRUE. , IBRION=8), and the resulting IR-spectra are fully consistent.

For the electronic band structure and effective mass calculations we have included spin-orbit coupling (SOC) effects, used a 600 eV cutoff, a 10^{-6} eV electronic convergence criterion, and evaluated projectors in real space (LREAL = Auto). All other parameters were the same as above. Plotting of the electronic bands and extraction of effective masses was done using the sumo package[18].

Phonon band structures and IR spectrum

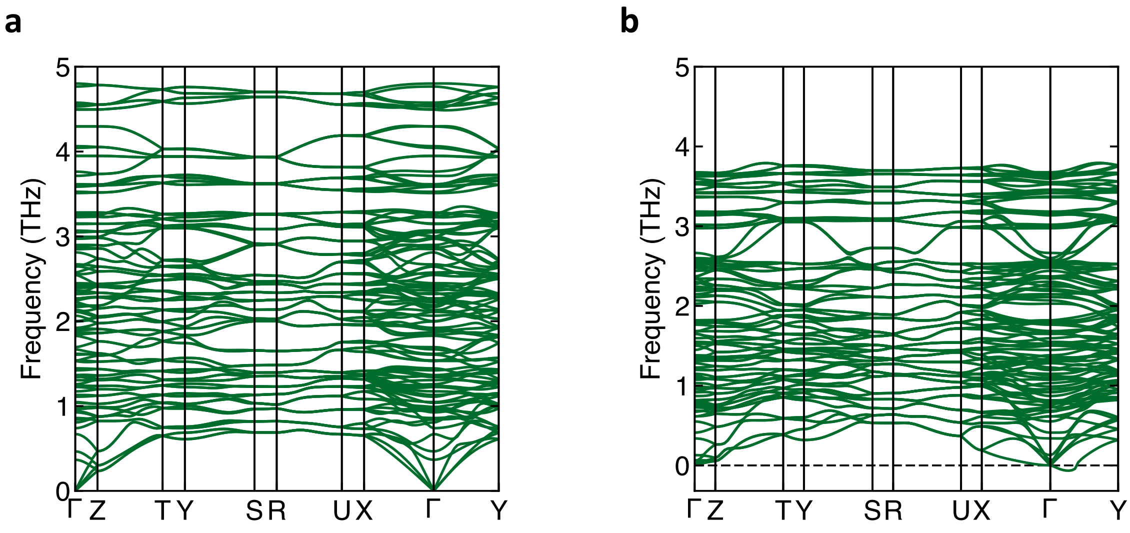

Figure S10: Low-frequency parts of the phonon dispersion relations of the (a) LT and (b) HT phases. The small imaginary mode near the Γ-point in the HT phase is likely a numerical artifact. In both phases, the Γ -Z corresponds to the out-of-plane direction while the Γ -X and Γ -Y corresponds to directions along the shorter and longer in-plane lattice vectors, respectively.

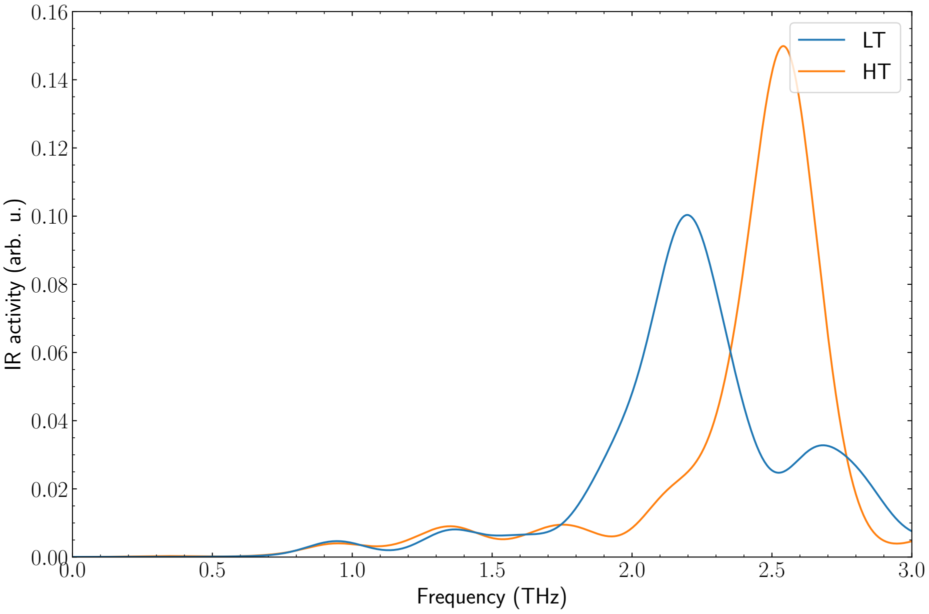

Figure S11: Comparison of the low frequency part of the calculated IR-spectrum for the n=1 LT and HT phases.

Directionally resolved IR spectrum

We calculated a directionally resolved IR-spectrum by projecting the mode-effective charges on a unit-vector initially along the direction of the (a) shortest lattice vector, summing over all modes, and then letting this unit-vector rotate in-plane.

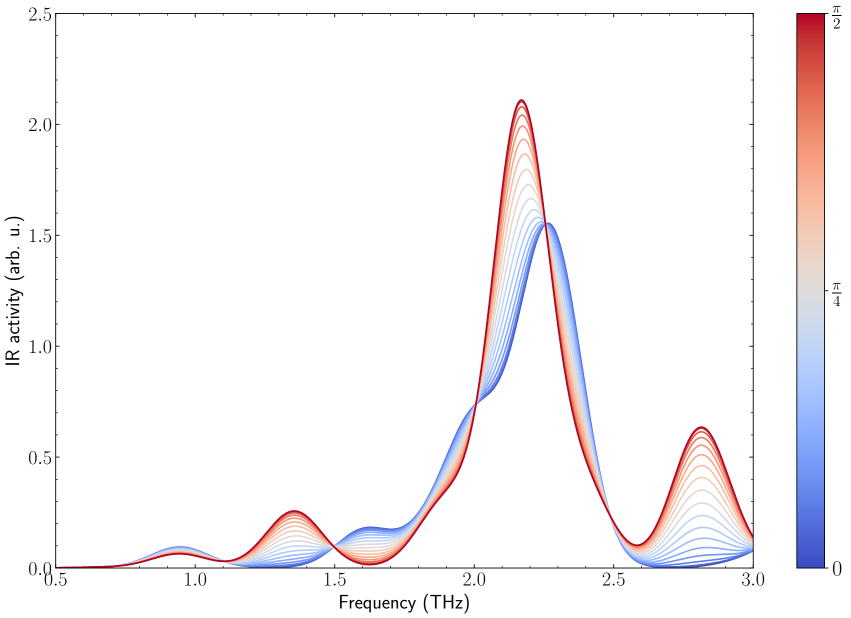

Figure S12: Directionally resolved IR-spectrum. The color scale gives the direction of the external field, in terms of positive in-plane rotation away from the shortest (a) in-plane lattice vector.

Electronic band structure

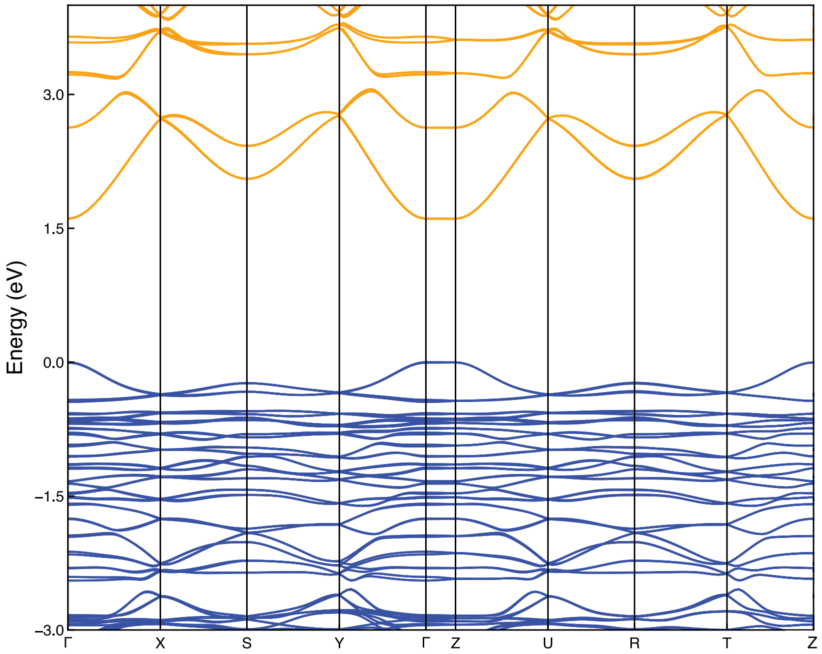

Figure S13: Electronic bandstructure, calculated using PBE+TS including SOC, of the n=1 LT phase. Parabolic fits to the band edges yields the following effective masses (in bare electron masses). Electrons: Γ-X: 0.249 , Γ -Y: 0.226. Holes: Γ -X: 0.396, Γ -Y: 0.397. Γ -Z corresponds to the out-of-plane direction while Γ -X and Γ -Y corresponds to directions along the shorter and longer in-plane lattice vectors, respectively.

Electron-phonon interaction and directional photoconductivity

To calculate the electron-phonon coupling strength, and its resulting impact on electron conductivity, we followed the same method we developed in reference[19]. In short, this is a matter of retaining the information about the phonon eigenvector, and using this information to calculate the crystal-plane dependent dielectric constants. These can then be used, together with the anisotropic effective mass, to calculate a crystal-plane dependent alpha, the Fröhlich dimensionless electron-phonon coupling constant. With this anisotropic alpha, you can then

immediately use the standard Feynman variational approach to modelling polarons and the associated FHIP mobility theory, which is by it's nature 1D.

In the standard isotropic approach to calculating an alpha, the individual crystal eigenmodes are averaged other (resulting in a pre-factor of 1/3 in the dielectric function). This is generally quite a good approximation for bulk materials, as there are typically similar polar modes in all 3 crystal directions. In 2D materials, this assumption breaks down quite considerably.

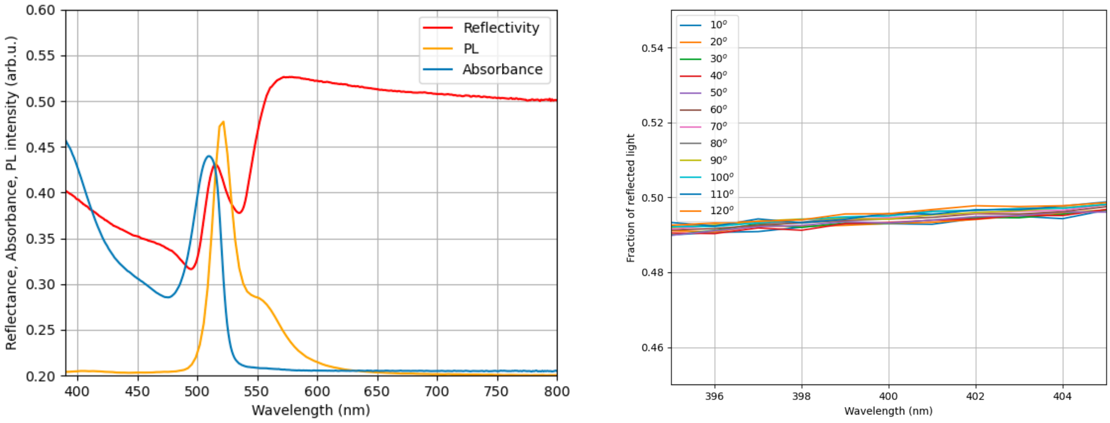

Figure S14: The left figure shows the comparison between the absorption of a spincoated film of n = 1 BAPI, and the reflectivity and photoluminescence of a single crystalline n = 1 BAPI sample. The right figure shows the measured fraction of reflected light around the excitation wavelength used for the OPTP experiments (400 nm) using linearly polarized light. The indicated angle is w.r.t. the transmission axis of the linear polarizer, the samples were rotated for these experiments. Little to no anisotropy of the reflectivity (and by Kramers Kronig relations the absorbance) can be observed.

The reflectivity spectra in Figure 1(c) of the main text for all different layer thicknesses show two features. A transition at higher energy, which corresponds to the excitonic absorption line, and a sub-bandgap feature at longer wavelengths, both of which are indicated by vertical

dashed lines in the spectra. The energy-spacing is 190 meV, 178 meV, 163 meV and 130 meV for n = 1,2,3,4 respectively. The sub-bandgap absorption line, which can also be observed in emission, has been ascribed to various origins, from states localized at the edges of crystals to magnetic dipole emission, and are discussed in references [20–23] of the main text.

SI7 – Anisotropy ratio determination from THz spectroscopy

We have estimated the anisotropy in photoconductivity, displayed in Figure 5(d), in three different ways.

1. Firstly, we averaged the real part of the OPTP signals from 9.6 to 10.4 ps [the region indicated by the blue shaded region in Figure 4(a-d)]. We then divided the average in the direction perpendicular direction by the average in the direction parallel to the 1 THz transition dipole moment as our first anisotropy value.

2. Secondly, we avaraged the real part of the photoconductivity spectra shown in Figure 4(e-h). Again, we then divided the average in the perpendicular direction by the average in the direction parallel to the 1 THz transition dipole moment as our second anisotropy value.

3. Lastly, we divided the scattering times, obtained from the Drude first to the photoconductivity spectra shown in Figure (e-h), in the perpendicular direction by the scattering time in the direction parallel to the 1 THz transition dipole moment as our third anisotropy value.

These results are shown in Figure 5(d). The red dots are the above values of the direction parallel divided by themselves, and as such are at a value of one, which serve as an indication to the other datapoints.

SI8 - Supplementary references

(1) Wang, K.; Congcong Wu, #; Yang, D.; Jiang, Y.; Priya, S. Quasi-Two-Dimensional Halide Perovskite Single Crystal Photodetector. *ACS Nano* **2018**, *12*, 33. https://doi.org/10.1021/acsnano.8b01999.

(2) Stoumpos, C. C.; Cao, D. H.; Clark, D. J.; Young, J.; Rondinelli, J. M.; Jang, J. I.; Hupp, J. T.; Kanatzidis, M. G. Ruddlesden-Popper Hybrid Lead Iodide Perovskite 2D Homologous Semiconductors. *Chemistry of Materials* **2016**, *28* (8), 2852–2867. https://doi.org/10.1021/acs.chemmater.6b00847.

(3) Glover, R. E.; Tinkham, M. Conductivity of Superconducting Films for Photon Energies between 0.3 and $40k{T}_{c}$. *Phys. Rev.* **1957**, *108* (2), 243–256. https://doi.org/10.1103/PhysRev.108.243.

(4) Zhao, D.; Hu, H.; Haselsberger, R.; Marcus, R. A.; Michel-Beyerle, M. E.; Lam, Y. M.; Zhu, J. X.; La-O-Vorakiat, C.; Beard, M. C.; Chia, E. E. M. Monitoring Electron-Phonon Interactions in Lead Halide Perovskites Using Time-Resolved Thz Spectroscopy. *ACS Nano* **2019**, *13* (8), 8826–8835. https://doi.org/10.1021/acsnano.9b02049.

(5) Jepsen, P. U. Phase Retrieval in Terahertz Time-Domain Measurements: A "How to" Tutorial. *Journal of Infrared, Millimeter, and Terahertz Waves 2019 40:4* **2019**, *40* (4), 395–411. https://doi.org/10.1007/S10762-019-00578-0.

(6) Pupeza, I.; Koch, M.; Wilk, R. Highly Accurate Optical Material Parameter Determination with THz Time-Domain Spectroscopy. *Optics Express, Vol. 15, Issue 7, pp. 4335-4350* **2007**, *15* (7), 4335–4350. https://doi.org/10.1364/OE.15.004335.

(7) Němec, H.; Kadlec, F.; Kužel, P. Methodology of an Optical Pump-Terahertz Probe Experiment: An Analytical Frequency-Domain Approach. *The Journal of Chemical Physics* **2002**, *117* (18), 8454. https://doi.org/10.1063/1.1512648.

(8) Kresse, G.; Hafner, J. Ab Initio Molecular Dynamics for Liquid Metals. *Phys. Rev. B* **1993**, *47* (1), 558–561. https://doi.org/10.1103/PhysRevB.47.558.

(9) Kresse, G.; Furthmüller, J. Efficiency of Ab-Initio Total Energy Calculations for Metals and Semiconductors Using a Plane-Wave Basis Set. *Computational Materials Science* **1996**, *6* (1), 15–50. https://doi.org/10.1016/0927-0256(96)00008-0.

(10) Kresse, G.; Furthmüller, J. Efficient Iterative Schemes for Ab Initio Total-Energy Calculations Using a Plane-Wave Basis Set. *Phys. Rev. B* **1996**, *54* (16), 11169–11186. https://doi.org/10.1103/PhysRevB.54.11169.

(11) Blöchl, P. E. Projector Augmented-Wave Method. *Phys. Rev. B* **1994**, *50* (24), 17953–17979. https://doi.org/10.1103/PhysRevB.50.17953.

(12) Perdew, J. P.; Burke, K.; Ernzerhof, M. Generalized Gradient Approximation Made Simple. *Phys. Rev. Lett.* **1996**, *77* (18), 3865–3868. https://doi.org/10.1103/PhysRevLett.77.3865.

(13) Tkatchenko, A.; Scheffler, M. Accurate Molecular Van Der Waals Interactions from Ground-State Electron Density and Free-Atom Reference Data. *Phys. Rev. Lett.* **2009**, *102* (7), 073005. https://doi.org/10.1103/PhysRevLett.102.073005.

(14) Menahem, M.; Dai, Z.; Aharon, S.; Sharma, R.; Asher, M.; Diskin-Posner, Y.; Korobko, R.; Rappe, A. M.; Yaffe, O. Strongly Anharmonic Octahedral Tilting in Two-Dimensional Hybrid Halide Perovskites. *ACS Nano* **2021**, *15* (6), 10153–10162. https://doi.org/10.1021/ACSNANO.1C02022.

(15) Togo, A. First-Principles Phonon Calculations with Phonopy and Phono3py. *J. Phys. Soc. Jpn.* **2023**, *92* (1), 012001. https://doi.org/10.7566/JPSJ.92.012001.

(16) Togo, A.; Chaput, L.; Tadano, T.; Tanaka, I. Implementation Strategies in Phonopy and Phono3py. *J. Phys.: Condens. Matter* **2023**, *35* (35), 353001. https://doi.org/10.1088/1361-648X/acd831.

(17) Skelton, J. M.; Burton, L. A.; Jackson, A. J.; Oba, F.; Parker, S. C.; Walsh, A. Lattice Dynamics of the Tin Sulphides SnS2, SnS and Sn2S3: Vibrational Spectra and Thermal Transport. *Phys. Chem. Chem. Phys.* **2017**, *19* (19), 12452–12465. https://doi.org/10.1039/C7CP01680H.

(18) Ganose, A. M.; Jackson, A. J.; Scanlon, D. O. Sumo: Command-Line Tools for Plotting and Analysis of Periodic *ab Initio* Calculations. *Journal of Open Source Software* **2018**, *3* (28), 717. https://doi.org/10.21105/joss.00717.

(19) Guster, B.; Melo, P.; Martin, B. A. A.; Brousseau-Couture, V.; de Abreu, J. C.; Miglio, A.; Giantomassi, M.; Côté, M.; Frost, J. M.; Verstraete, M. J.; Gonze, X. Fröhlich Polaron Effective Mass and Localization Length in Cubic Materials: Degenerate and Anisotropic Electronic Bands. *Physical Review B* **2021**, *104* (23), 235123. https://doi.org/10.1103/PHYSREVB.104.235123/FIGURES/10/MEDIUM.
(20) Guo, S.; Li, Y.; Mao, Y.; Tao, W.; Bu, K.; Fu, T.; Zhao, C.; Luo, H.; Hu, Q.; Zhu, H.; Shi, E.; Yang, W.; Dou, L.; Lü, X. Reconfiguring Band-Edge States and Charge Distribution of Organic Semiconductor–Incorporated 2D Perovskites via Pressure Gating. *Science Advances* **2022**, *8* (44), eadd1984. https://doi.org/10.1126/sciadv.add1984.
(21) Shi, E.; Deng, S.; Yuan, B.; Gao, Y.; Akriti; Yuan, L.; Davis, C. S.; Zemlyanov, D.; Yu, Y.; Huang, L.; Dou, L. Extrinsic and Dynamic Edge States of Two-Dimensional Lead Halide Perovskites. *ACS Nano* **2019**, *13* (2), 1635–1644. https://doi.org/10.1021/acsnano.8b07631.
(22) Lu, J.; Zhou, C.; Chen, W.; Wang, X.; Jia, B.; Wen, X. Origin and Physical Effects of Edge States in Two-Dimensional Ruddlesden-Popper Perovskites. *iScience* **2022**, *25* (6), 104420. https://doi.org/10.1016/j.isci.2022.104420.
(23) DeCrescent, R. A.; Venkatesan, N. R.; Dahlman, C. J.; Kennard, R. M.; Zhang, X.; Li, W.; Du, X.; Chabinyc, M. L.; Zia, R.; Schuller, J. A. Bright Magnetic Dipole Radiation from Two-Dimensional Lead-Halide Perovskites. *Science Advances* **2020**, *6* (6). https://doi.org/10.1126/SCIADV.AAY4900/SUPPL_FILE/AAY4900_SM.PDF.
